# How to assess? Perceptions and preferences of undergraduate medical students concerning traditional assessment methods

**DOI:** 10.1186/s12909-020-02239-6

**Published:** 2020-09-17

**Authors:** Anita Holzinger, Stefan Lettner, Verena Steiner-Hofbauer, Meskuere Capan Melser

**Affiliations:** 1 Research Unit for Curriculum Development, Teaching Center/Medical University of Vienna, Spitalgasse 23, Bauteil 87, A-1090 Vienna, Austria; 2 University Clinic of Dentistry/Medical University of Vienna, Vienna, Austria

**Keywords:** Traditional assessments, Perceptions of undergraduate medical students, Preferences

## Abstract

**Background:**

Medical students’ perception of traditional assessments have an important impact on their approaches to learning. Even though these assessment formats such as Multiple-Choice Question (MCQ), Short Answer Question (SAQ) or oral examinations, are frequently used in medical curricula, only little is known about student’s perceptions of these assessments. The objective of this study was to assess perceptions and preferences of undergraduate medical students concerning traditional assessment formats.

**Methods:**

The study was conducted at the Medical University of Vienna. Attitudes of 2nd year undergraduate medical students towards traditional assessment formats, and their relation to students’ learning, and students’ attitude towards objectivity, was inquired using a self-developed questionnaire.

**Results:**

459 students participated in this study. MCQs examinations were the most preferred assessment format and were chosen as the most objective format. Most students agreed that oral examinations are more appropriate for achieving long-term knowledge. Female students showed higher preference for oral examinations than male students. Students would prefer an assessment mix of 41.8% MCQs, 24.0% oral examinations, and 9.5% SAQs, if they were free to choose the assessment tools.

**Conclusion:**

Students prefer MCQ format over SAQs/oral examinations. Students’ subjective perception of the importance of gaining long-term knowledge through an assessment has no influence on their assessment preference.

## Background

Assessments in higher education have several functions, including grading, evaluation of student achievement and supporting student’s learning. How to assess in medical education is still the subject of many controversial discussions. Traditional assessment methods like traditional or structured oral examinations, Multiple Choice Question (MCQ) formats, and Short Answer Question (SAQ) formats have been widely criticised for different reasons. Even though MCQ format is an efficient assessment method for universities with large student cohorts - it is objective, transparent, economic, and enables to measure students’ knowledge up to competence level - this format can danger the cumulative learning and long-term retention of medical knowledge [1, 2]. Besides, writing a higher order thinking MC question can be challenging for item writers [3, 4]. SAQ format in turn, can test a wide range of topics with high reliability [5] but, the evaluation of results is time consuming and is therefore not feasible as an instrument for testing a large number of students [6]. Finally, oral examinations enable the instructors to measure for example clinical competence of students or to judge students associative and strategic thinking. The use of oral examinations has been criticized because of low reliability, low validity, and examiners bias [7, 8]. Additionally, Cees van der Vleuten stated that the utility of an assessment method depends on its reliability, validity, costs, educational impact and its acceptability [9, 10].

During the last decade, the investigation of students’ assessment preference has gained increased attention due to understanding factors that drive the learning process and its outcomes. Research findings point out that students’ perceptions of assessment have considerable influence on students’ approaches to learning and studying [11]. Vice versa, students’ assessment perception influences their evaluation of the lecturers and lectures [11, 12]. Furthermore, students’ preferences of assessment reflect their perception of learning environment, their learning conceptions, and their approaches to learning [11, 13]. When students asked for their perception of learning approaches by using different assessment techniques, three main approaches are identified [11]. Surface approaches intend to address an association between routine memorisation and procedural problem solving [14–16]. In contrast, deep approaches to learning lead from an intention to understanding and are associated with active conceptual analysis [15, 16]. The third one, the strategic or achieving approach, described as intention to achieve the highest possible grades by using well-organised and conscientious study methods [14, 15]. As assessment is one of the defining features of the students’ approaches to learning [17–19], Marton and Saljo investigated the relationship between assessment and students’ approaches to learning, and found that students’ preferred assessment requirements are strongly related with their approaches to learning [18]. Similarly, Ramsden found that students often explained surface approaches or negative attitudes in terms of their experiences of inappropriate forms of assessment [19, 20]. Entwistle and Tait also found that the types of assessment do have influence on the way how students learn [21]. For example, multiple choice question formats push students towards surface approaches, while open, essay type questions encourage them to pursue a deep level of understanding [21], and a long-term knowledge achieving. Students with surface learning approaches mostly prefer multiple choice tests viewing it as being easier to prepare, easier to take, less complex, more interesting, less tricky, and fairer [22]. Also, students’ intention for achieving higher relative scores leads them to develop positive attitudes towards MCQ format [14, 15]. And similarly students with poor learning skills, who have low confidence in their academic ability, prefer MCQ format over other types of traditional assessments [23]. On the other hand, studies showed that students with good learning skills and high competence in their academic ability tend to prefer the essay type of assessment to the MCQ format [23]. Overall, assessment formats seem to have a considerable impact on students’ approaches to learning; and students’ perception of assessment and their approaches to learning seem to be strongly related [11].

As far as we know, there are only few studies that studied the perception of medical students towards traditional assessment tools. Findings of these studies present that the majority of medical students prefer MCQ format as summative assessment over open-ended or essay evaluations [24–26]. Oral examination are not very popular among medical students because of their lack of objectivity and examiner bias [24].

Even though deep learning should be an achievable learning approach at higher medical education, assessments like MCQ formats, which force students to surface approach, are widely spread examination tools. This holds also for the Medical University of Vienna where examinations consist of 45% MCQ formats, 15% SAQ formats and 40% other assessments such as oral examinations, OSCE, key-feature questions, mini-clinical evaluation exercise (mini-Cex), direct observation of procedural skills (DOPs) and self-assessment. Currently efforts are being made to alter the examinations, particularly changes in the mix of the various assessment formats are being discussed. The intended reforms will be guided by recent results of research in the didactics of medical education. To facilitate the implementation of changes, also students’ views on this issue will be taken into consideration. Until now only a few studies on students’ perceptions of examination modalities have been published, showing a preference for the MCQ format [24–26]. We therefore decided to carry out a survey aimed at exploring 3rd semester medical and dental students’ opinions on summative integrated assessment formats such as MCQ, SAQ, and traditional oral examinations, with which students are familiar from the beginning of their medical education. First, we were interested in students’ preferences regarding these assessment formats. Second, we aimed at finding out students’ views on assessment characteristics like difficulty, length, and content of assessment. We also wanted to understand how the assessment formats affect students’ intention to achieve long-term medical knowledge. Third, we wanted to investigate students’ perception of the objectivity of the various assessment formats. Additionally, we explored students’ views about how variable the usage of assessment methods should be, if they were free to choose.

## Methods

### Questionnaire

In order to gain insight into the attitudes of medical and dental students towards traditionally used assessment formats, the Research Unit in cooperation with the Assessment & Skills Department of the Teaching Center kindly asked the students to participate in this study. A self-created questionnaire was handed out to third semester medical students (*n* = 459) during mandatory courses. 194 female students and 251 male students (44 dental students and 391 medical students) volunteered to fill out the anonymous questionnaire. All students signed a declaration of consent. The study was approved by the data protection and clearing committee of the MedUni Wien.

The questionnaire was based on recently used summative assessment methods, their long-lasting effect on medical students learning, and their objectivity, difficulty, content and duration. Along with sociodemographic information such as sex (11 students did not answer this question) and education (dental or medical medicine, 24 students did not answer this question) the questionnaire included 5 different parts that addressed opinion, expectation, aspiration of undergraduate medical and dental students on various assessment methods used in medical education as well as attitudes of medical students towards concept of teaching/learning activity.

The questionnaire measured levels of agreement on a four level ordinal scale ranging from “strongly disagree” to “strongly agree”. Questions related to extent criteria such as difficulty, duration and content of assessment measured levels of opinion on a three level ordinal scale. Further specific question regarding the mix of assessment methods in percentage was included to seek more detail about the assessment preference of students if they were free to choose. In total, 84.9% of all questions were completed.

### Statistical methods

Data was entered using SPSS version 24. Statistical analysis were done by using R 3.6.0 [27]. Mean values and bootstrapped 95% confidence intervals were calculated for all results. Graphics were created using ggplot 2 [28]. Percentage responses to mixed assessment questions which did not sum up to 100% were scaled accordingly. The correlation between students’ perception and assessment methods was analysed by using spearman’s rho.

The cumulative link mixed model has been used to analyse the students’ assessment preference [29]. Students ID has been chosen as a random factor, assessment methods, long-term learning, and objectivity of assessment and students’ satisfaction have been used as fixed explanatory factors.

## Results

**Fig. 1:**
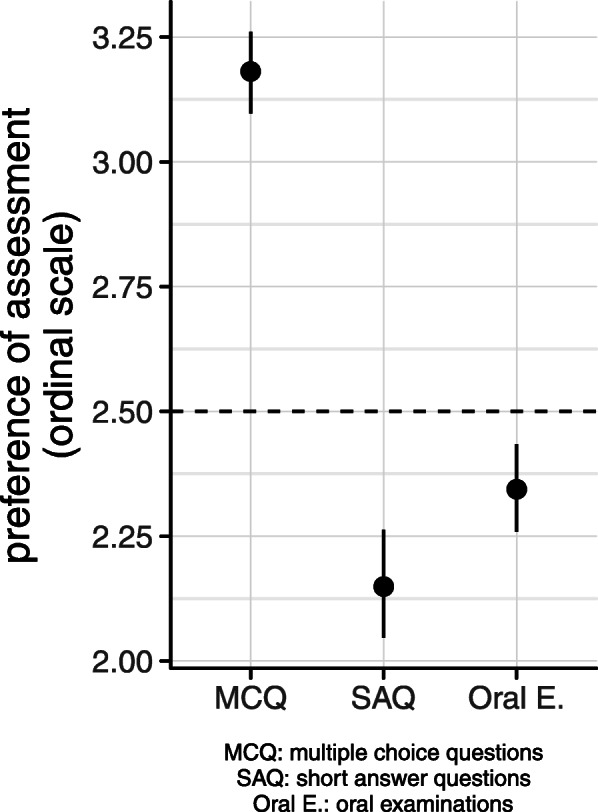
Students’ preferences regarding assessment methods

**Fig. 2:**
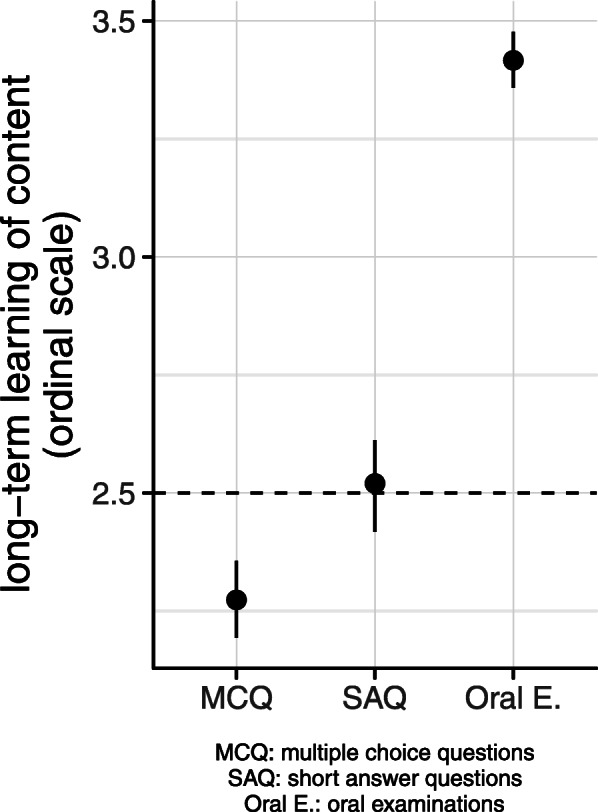
Students’ views on the appropriateness of the various assessment methods for achieving long-term knowledge

Most of the students preferred MCQs over oral examinations and short answer questions (mean 3.18, SD ± 0.89 for MCQs; mean 2.34, SD ± 0.94 for oral examination; and mean 2.15, SD ± 0.89 for SAQs) (Fig. 1). Most students found oral examinations more appropriate for achieving long-term knowledge than MCQs and SAQs (mean 3.42, SD ± 0.65; mean 2.27, SD ± 0.87; and mean 2.52, SD ± 0.83) (Fig. 2).

**Fig. 3:**
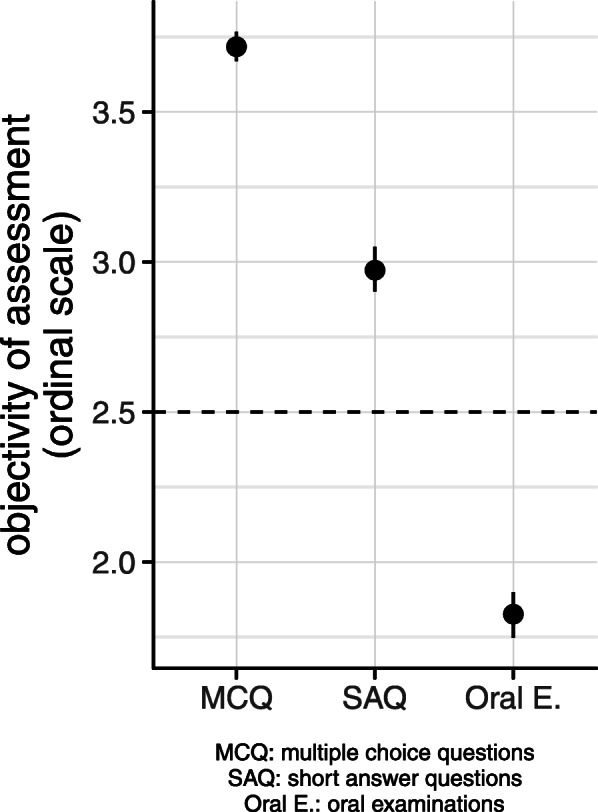
Students’ appraisal of the objectivity of the various assessment methods

Additionally, oral examinations were considered less objective than SAQs and MCQs (mean 1.83, SD ± 0.79; mean 2.97, SD ± 0.73; and mean 3.72, SD ± 0.53). The majority of students judged MCQs as an objective tool (Fig. 3). In addition, significant gender differences emerged with respect to the oral examination format, with female students having more favourable attitudes than male students. The correlation coefficient of students’ perception towards oral examination is *r* = 0.60 for female and *r* = 0.34 for male students.

**Fig. 4:**
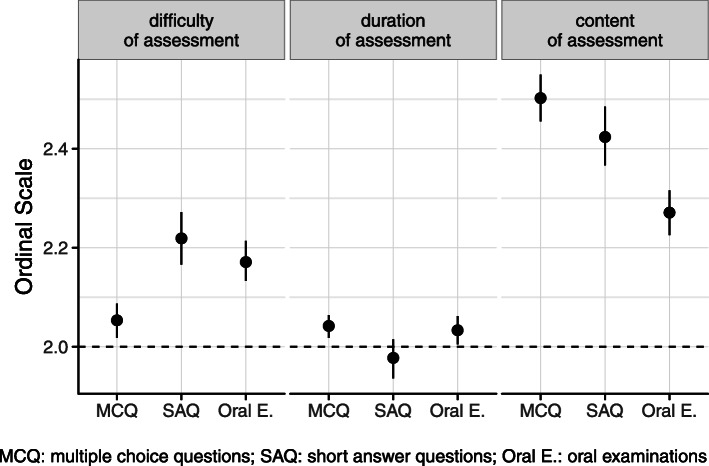
Students’ judgment of the difficulty, duration and content of the various assessments

Students’ response was obtained regarding extent criteria such as difficulty of assessment, duration of assessment and content of assessment. The difficulty of assessments was rated by students appropriately. There was no significant difference between MCQs, SAQs and oral examinations (mean 2.05, SD ± 0.36; mean 2.22, SD ± 0.45; and mean 2.17, SD ± 0.41) (Fig. 4). Student’s rating of assessment duration was reasonable and there was no difference between various methods (mean 1.98, SD ± 0.32 for SAQs and mean 2.03, SD ± 0.26 for oral examination) (Fig. 4). The students were also asked about the quantity of examination material, and most students found that the content of assessments are a lot to learn (mean 2.42, SD ± 0.50 for SAQs; mean 2.27, SD ± 0.46 for oral examinations).

Were students free to choose, they would prefer a mix of 41.8% MCQs, 24.0% oral examinations, 9.5% SAQs, and 24.7% other assessment methods such as OSCE and open-ended questions. Most students choose MCQs as the main assessment format followed by oral examinations.

**Fig. 5:**
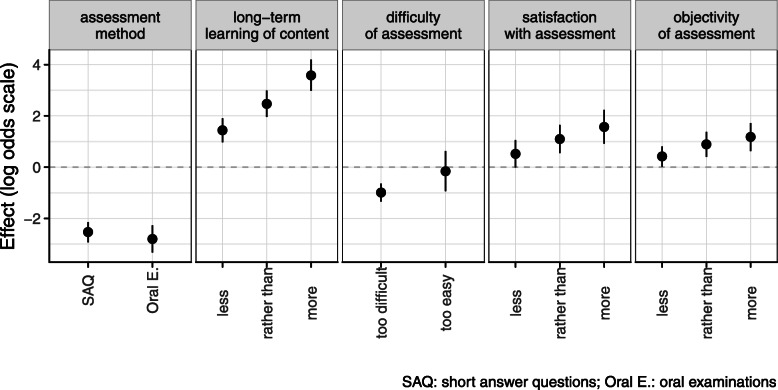
Graphic representation of Table 1

Modelling assessment preference of students showed that in general, MCQs were highly preferred over SAQs (log odds − 2.53) and oral examinations (logs odds − 2.80). This preference was mainly modified by student appraisal of long-term learning (log odds increase by 3.58 from negative to positive appraisal), followed by general satisfaction (increase by 1.57 from negative to positive) and objectivity of assessment methods (increase by 1.18 from negative to positive). In comparison with other factors, the difficulty of assessment was perceived least important (decrease by 0.83 from too easy to too difficult) (Table 1, Fig. 5). All of the above-mentioned factors were significant (*p* < 0.001).

**Table 1:** Estimates for log odds and corresponding confidence intervals in the mixed logistic ordinal model

Assessment methods	Short Answer Questions (SAQs)	−2.53	−2.93	− 2.14
	Oral Examinations	−2.8	−3.33	−2.27
Long-term Learning	less	1.44	0.97	1.91
	rather than	2.47	1.96	2.99
	more	3.58	2.98	4.2
Difficulty of Assessment	too difficult	−0.99	−1.34	−0.64
	too easy	−0.16	−0.94	0.63
Satisfaction of Students	less	0.52	−0.01	1.06
	rather than	1.1	0.55	1.65
	more	1.57	0.92	2.23
Objectivity of Assessment	less	0.42	0.02	0.81
	rather than	0.89	0.4	1.38
	more	1.18	0.63	1.72

## Discussion

Aim of this study is to assess students’ perception of traditional assessment methods and to examine the impact of students’ learning activity on their preferences. This study also aimed at understanding the relation between students’ perception of assessment and objectivity in assessment.

The results show that students perceive MCQ format more favourable than SAQ format and oral examinations. Students’ higher perceived objectivity and general satisfaction give preference to this assessment format.

MCQs have been widely used for summative assessment in undergraduate medical education because of their convenient standardization, efficient testing for large classes and a broad sampling of knowledge [30]. Well-constructed MCQs allow the evaluation of taxonomically higher-order cognitive skills such as application of knowledge, interpretation, and synthesis rather than the test of recall of isolated facts [31]. Our study showed that most of the students prefer MCQ format to SAQs and oral examinations on the grounds that this examination seems easier to prepare for and easier to pass, which is in agreement with other studies [24, 25, 32].

SAQs have been commonly used in examinations to assess the basic knowledge and understanding of a topic. SAQs tend to test higher-level thinking and assesses mainly knowledge, comprehension, and some application if they can incorporate clinical scenarios. In this study, students evaluated SAQs less favourably. Most students found that SAQs were more difficult than MCQs and oral examinations. On the other hand, studies showed that the attitudes towards each of these assessment formats correlated with students’ learning-related processes of the cognitive and affective aspects. Students with good learning skills, who have high confidence in their academic ability, tend to prefer SAQ type of assessment over the multiple choice type of assessment [23]. Nevertheless, these results do not apply to our students. In their view, SAQs do not encourage long-term retention of medical knowledge, and are less objective in comparison to MCQ format. Our students also found that oral examinations invoke long-term retention of medical knowledge more than MCQs and SAQs and are more preferred than SAQ format.

Furnham et.al. demonstrated that multiple choice questions are preferred by bright, and less open candidates, and oral exams are better suited for stable, low conscientious students with a deep learning style [33]. It has been also suggested that students with a deep study approach tend to prefer essay-type questions, while students with a surface study approach tend to prefer multiple choice formats [13]. In contrast to this study, in our model, we tried to understand the factors that influence the assessment preference of students in undergraduate medical education, and showed that long-term retention has not the greatest influence on exam preference., Students using deep approaches to learning for examinations still tend to prefer MCQs over oral examinations, despite the fact that they found oral examinations encouraging long-term retention of medical knowledge. Our conclusion is that MCQ format is easier to learn and the structure of MCQ formats allows an economical approach to learning.

Some studies report gender effects on students’ preferences of assessments. In this study, we also observed gender differences, with female students having more favourable attitudes towards oral examinations than males. Birenbaum and Feldman demonstrated that MCQ formats are considered less favourable by females than males in comparison to essay examinations [23]. Other studies also showed that gender causes high differences in preferences of written exams [34]. The reasons could be 1) the relationship between preference and personal dimension of risk taking [35]; for example: females were more reluctant than males to guess on MCQs and were more likely to leave items blank; or 2) the relation between personality traits and preference of assessment methods. Another possible explanation could be that oral examinations may bepreferred by women, because they converse with more questions and develop connections with others (in this case “examiners”) through talking in contrast to men, who prefer direct statements and have a “get it done” approach [36].

As concerns the preferred mix of assessment formats, we found that the share of MCQ format examinations in our current curriculum meets students’ expectations. Besides, students prefer oral examinations or other alternative assessment formats over SAQ examinations. Overall, the satisfaction of students with the various assessment methods in the curriculum determines how successful they will be in using their medical knowledge in the future.

Assessment drives learning in all forms of education including medical education [37]. Therefore, it is necessary to study students’ attitudes towards different assessment formats before implementing a new curriculum. Students will be more motivated and hence even would perform better if they are assessed through methods of their choice.

## Conclusion

In conclusion, our results provide an additional information and evidence on students’ perceptions about traditional assessment formats, and students’ satisfaction about gaining long-term knowledge through these assessments, which can be an important guide to improve their assessment practices, and to achieve a higher quality of learning and education.

## Limitations

Only the opinion of students who were in second year of undergraduate medical education could be obtained. We also did not try to evaluate student learning style (surface approaches vs. deep approaches) and student performance in respect to distribution rates, which would have been related to our study. Due to the small number of dental students a comparison with medical students was not feasible.

## Data Availability

The questionnaire used during this study is available from the corresponding author on reasonable request.

## Abbreviations

MCQ: Multiple choice question
SAQ: Short answer question
OSCE: Objective structured clinical examination
DOPs: Direct observation of procedural skills
Mini-CEX: Mini-clinical evaluation exercise
